# PCSK6 in Pathophysiology and Its Potential Involvement in Pulmonary Fibrosis

**DOI:** 10.3390/molecules31213931

**Published:** 2026-11-01

**Authors:** Qian Tian, C. Arnold Spek, Jan Willem Duitman

**Affiliations:** 1 Department of Experimental Immunology, Amsterdam UMC Location University of Amsterdam, 1105 AZ Amsterdam, The Netherlands; 2 Amsterdam Institute of Immunology & Infectious Disease, Amsterdam UMC Location University of Amsterdam, 1105 AZ Amsterdam, The Netherlands; 3 Center for Infection and Molecular Medicine, Amsterdam UMC Location University of Amsterdam, 1105 AZ Amsterdam, The Netherlands; c.a.spek@amsterdamumc.nl; 4 Laboratory of Experimental Oncology and Radiobiology, Amsterdam UMC Location University of Amsterdam, 1105 AZ Amsterdam, The Netherlands; 5 Department of Pulmonary Medicine, Amsterdam UMC Location University of Amsterdam, 1105 AZ Amsterdam, The Netherlands

**Keywords:** proprotein convertase subtilisin/kexin type 6, proteases, substrates, pathophysiology, pulmonary fibrosis

## Abstract

Proprotein convertase subtilisin/kexin type 6 (PCSK6) is a proteolytic enzyme that may act on a broad spectrum of substrates, including growth factors, extracellular proteases, adhesion molecules, protease activated receptor 1, the prorenin receptor, and Corin. As a consequence of its broad-spectrum substrate activity, PCSK6 is implicated in a wide range of pathophysiological processes such as cardiovascular disease, neurological disorders, arthritis and cancer. Of special interest, PCSK6 has recently been shown to be highly expressed in patients with idiopathic pulmonary fibrosis (IPF) and PCSK6 has been associated with reduced survival of IPF patients, linking PCSK6 to pulmonary fibrosis for the first time. Of note however, PCSK6 levels in plasma were not associated with respiratory outcomes in IPF patients and higher PCSK6 levels were even associated with a lower hazard of 10% FVC decline in non-IPF ILD patients. In this review, we summarize the molecular biology of PCSK6, including its structural features and its major (potential) substrates, and outline its pathophysiological roles across various diseases. Furthermore, we propose and discuss the potential involvement of PCSK6 in pulmonary fibrosis to guide future research.

## 1. Introduction

Proteolytic cleavage is an essential process in which latent precursor proteins are converted into their biologically active forms, a step indispensable for numerous physiological functions [1]. The proprotein convertase subtilisin/kexin (PCSK) family, characterized by its proteolytic activity, is capable of activating a wide range of proteins, including proteases, growth factors, and adhesion molecules [2]. In mammals, the PCSK family comprises nine secreted serine proteases, among which proprotein convertase subtilisin/kexin type 6 (PCSK6), also referred to as subtilisin-like proprotein convertase 4 (SPC4) or paired basic amino acid-cleaving enzyme 4 (PACE4), is a prominent member [3].

PCSK6 exhibits widespread expression in multiple human organs, such as the liver, spleen, gastrointestinal tract, pancreas, lung, heart, kidney, uterus, placenta, ovary, brain and neuroendocrine tissues [2,4]. Over the last decade, PCSK6 has emerged as a critical player in mediating distinct biological processes, such as organogenesis, tissue maintenance and repair, primarily through PCSK6’s ability to activate and modulate proteases, growth factors and extracellular matrix (ECM) proteins. Moreover, aberrant PCSK6 expression and activity have recently been shown to contribute to the pathogenesis of various diseases, including cancer, neurodegenerative disorders, cardiovascular disease, and musculoskeletal disorders.

Recently, two association studies showed that specific PCSK6 gene variants [5] and higher plasma PCSK6 levels were associated with decreased survival of idiopathic pulmonary fibrosis (IPF) patients [5,6]. In IPF, increased staining intensity in lung tissue, elevated transcript levels in peripheral blood and higher circulating plasma protein concentrations were all associated with reduced survival [5]. Moreover, PCSK6 variants were not associated with the risk for IPF development. Interestingly, in a mixed fibrotic ILD group, no associations between plasma PCSK6 levels and time to death, lung transplantation, respiratory hospitalization, and 10% FVC decline were observed [6]. Of note, higher PCSK6 was significantly associated with a lower hazard of 10% FVC decline in the non-IPF subgroup, raising the question of which underlying mechanism causes the IPF specific association. These results suggest that PCSK6 may be an IPF-specific prognostic biomarker for survival, and may have disease- and cell-type-specific effects that differ between IPF and other fibrotic ILDs. Nevertheless, additional studies are required to delineate PCSK6’s actual role and mechanistic effects in pulmonary fibrosis. To facilitate future research, this review integrates current knowledge of PCSK6, highlighting its molecular characteristics and diverse pathophysiological functions across disease contexts. In this review, we specifically focus on PCSK6, summarizing its molecular biology and physiological functions -particularly the activation of key substrates- and discussing the (potential) pathological implications in disease.

## 2. Molecular Biology of PCSK6

### 2.1. Structure and Activation

**Figure 1:**
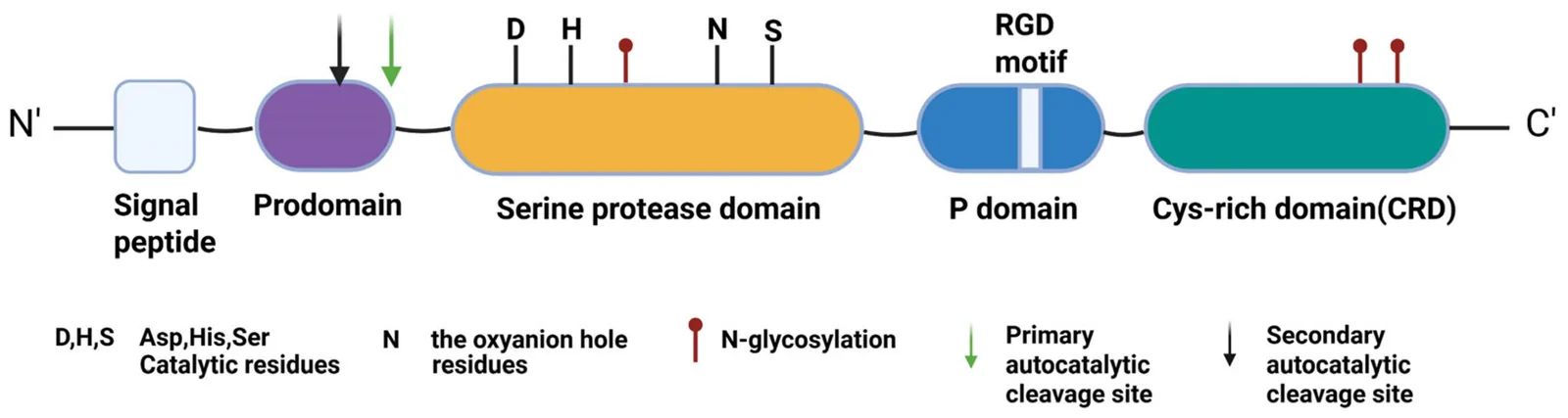
Protein structure of canonical PCSK6. Canonical PCSK6 contains multiple functionally different regions: the N-terminal signal peptide serves as a classical signal peptide, guiding the pre-protein into the endoplasmic reticulum (ER), where it is cleaved off by signal peptidase; the prodomain facilitates correct folding of PCSK6 within the ER by acting as an intramolecular chaperone; the serine protease domain contains the catalytic triad residues D (Asp), H (His), and S (Ser), as well as the oxyanion hole residue (N); the P domain associates with the serine protease domain to support correct folding and to stabilize PCSK6. The green and dark arrows designate two intramolecular autocatalytic cleavage sites essential for the maturation of PCSK6. Finally, three N-glycosylation sites are present within the protein as indicated.

The *PCSK6* gene, located on human chromosome 15 in close proximity to FURIN, encodes a protease for which several transcript variants and protein isoforms have been identified [7,8,9,10]. Canonical PCSK6 is synthesized as a zymogen comprising 969 amino acids that are organized into multiple domains, starting with a long signal peptide, followed by a prodomain (prosegment), a catalytic (serine protease) domain, a P domain and a Cysteine-rich domain (CRD), respectively (Figure 1) [11,12]. The PCSK6E isoform, which contains 975 amino acid residues, has been shown to differ from the canonical isoform in its C-terminal region, and to exhibit retarded secretion [13]. The PCSK6C isoform, lacking 11 amino acids at the end of the P domain, was shown to be retained in the endoplasmic reticulum (ER), thereby remaining an inactive zymogen that is not secreted [14]. Structural differences between PCSK6 isoforms could potentially affect PCSK6 localization, secretion, or substrate accessibility, indicating PCSK6 isoforms may not be functionally equivalent [13,14,15]. Similar to many other serine proteases, PCSK6 is produced as a precursor protein and undergoes a series of post-translational modifications before attaining its activity.

Based on the known function of the different domains and the results of biochemical experiments, the following steps are required to attain an enzymatically active mature protein. The first processing steps take place within the ER, where signal peptidase cleaves and removes the signal peptide. As an intramolecular chaperone, the N-terminal prodomain directs the proper folding of PCSK6 into its active conformation within the ER lumen, which enables subsequent intramolecular autocatalytic cleavage of the prodomain [3]. After this cleavage event, the prodomain remains bound to the serine protease domain to keep the enzyme inactive during secretion [16,17].

The P domain, containing a calcium-binding site, also interacts with the serine protease domain to ensure proper folding of the pro-protein and to maintain structural stability through hydrophobic patches [18]. In addition, the P domain helps to maintain the zymogen in an inactive state until prodomain cleavage [19]. Moreover, the P domain regulates intracellular trafficking through its RGD motif [20], and the P domain functions together with the CRD to promote cell surface attachment by binding to heparan sulfate proteoglycans (HSPGs) [21]. These HSPGs tether PCSK6 to the extracellular matrix at the cell surface. After secretion, PCSK6 undergoes an additional cleavage event at the cell surface, during which the prodomain is removed and the enzyme becomes fully active [4]. The CRD further facilitates interactions with cognate surface-anchored proteins, such as TIMPs. By positioning PCSK6 near certain cell compartments or extracellular structures, it also indirectly determines which substrates are accessible for cleavage [10,11].

### 2.2. Key Substrates

Proprotein convertases (PCs) including PC1/3, PC2, Furin, PC4, PCSK6, PC5/6 (PCSK5), and PC7 (PCSK7), cleave secretory precursors at canonical (R/K)-X_n_-(R/K)↓ motifs [22,23]. The mammalian PC family can be categorized according to their subcellular localization and physiological function. Furin, PCSK6, PC5/6 and PC7 are widely expressed across tissues and predominantly localize to the trans-Golgi network, endosomal system and cell surface, where they participate in constitutive secretory pathways [3,24,25]. Because members of the PC family recognize similar cleavage motifs and exhibit overlapping tissue expression, it is challenging to assess which specific precursor is processed by a given convertase [23]. Nevertheless, using computer modeling followed by peptide cleavage experiments, preferred substrate sequences have been determined for all different PCs showing a high preference for R-X-(R/K)-R↓ motifs [26]. Using similar approaches, numerous substrates have been identified as (potential) substrates of PCSK6, including growth factors, extracellular proteases, adhesion molecules, protease-activated receptor 1 (PAR-1), the prorenin receptor (PRR) and Corin (Table 1).

#### 2.2.1. Transforming Growth Factor-β (TGF-β) Family Ligands

TGF-β family ligands are synthesized as inactive precursors containing a pro-domain (called latency-associated peptide in case of canonical TGF-β) [27]. Proprotein convertase furin is known to cleave these precursors at basic amino acid cleavage sites, often containing R-X-K/R-R and RXXR motifs [28,29]. In embryos, PCSK6 deficiency leads to situs inversus, complex craniofacial abnormalities and cardiac malformations due to impaired activation of Nodal, a member of the TGF-β superfamily [30]. PCSK6 is also recognized as a critical factor for generating mature bone morphogenetic protein (BMP) from their inactive precursor proteins by endoproteolytic processing. In turn, BMP stimulation of human granulosa cells in vitro has been shown to suppress PCSK6 gene expression via a negative feedback system [31,32]. The growth differentiation factor 15 (GDF15) has likewise been identified as an efficient substrate of PCSK6 [33]. Because furin is well established as the protease responsible for producing mature TGF-β and knockdown of PCSK6 impacts TGF-β activation, it has been proposed that TGF-β may also be a substrate of PCSK6, given the substantial overlap in substrate specificity among members of the proprotein convertase family [1,34,35,36].

#### 2.2.2. Insulin-like Growth Factor-II (IGF-II) and Insulin Receptor B (IRB)

IGF-II, a member of the insulin-like growth factor (IGF) family, is a secreted growth factor. Evidence from IGF-II-deficient mouse models, as well as studies of patients carrying (epi)genetic abnormalities in IGF-II, has identified IGF-II as a key player involved in growth regulation, particularly normal fetal development, metabolism and tumorigenesis [37]. Pro-IGF-II undergoes extracellular activation through proteolytic processing by subtilisin-like proprotein convertases (SPCs). PCSK6 deficiency reduced IGF-II mRNA levels [38,39] and notably also inhibited the expression of the muscle-specific myosin light chain (MLC), an essential factor for myogenic differentiation [40]. Importantly, recombinant IGF-II treatment led to the recovery of MLC expression, suggesting that PCSK6 contributes to myogenic differentiation through the regulation of IGF-II [40].

The insulin receptor has two isoforms, IRA and IRB, generated through furin-mediated proteolytic cleavage of their propeptides. When furin-dependent maturation is impaired, the immature insulin receptor propeptides are transported to the cell surface, where PCSK6 can specifically enhance the maturation of the IRB isoform [41].

#### 2.2.3. Platelet-Derived Growth Factor (PDGF)-A/B and Vascular Endothelial Growth Factor (VEGF)-C

Besides IGF-II, PCSK6 may also contribute to the activation of growth factors like PDGF-A/B and VEGF-C, all of which are important in embryonic development and in the proliferative and migratory capacities of mesenchymal and vascular smooth muscle cells [42,43,44]. PDGF-A and PDGF-B are synthesized as proproteins containing paired basic residues. PDGF-A is cleaved at the RRKR(86↓) site, and this cleavage can be inhibited by furin-like proprotein convertase preprosegments (pps), including ppPCSK6 [45]. PCSK6 also cleaves pro-PDGF-B at the RGRR(81↓) site, a cleavage event involved in PDGF-B maturation and secretion. Furthermore, processing of the A and B chains is indispensable for the generation of functional PDGF-B dimers [46]. Treatment of megakaryoblastic cells with PDGF-BB increased PCSK6 expression, suggesting an elevated demand for processing activity and indicating a regulatory feedback interaction between PDGF-BB and PCSK6 [47].

VEGF-C is also synthesized as an inactive precursor protein that requires proteolytic cleavage to generate its mature active form [48]. Furin, PC5 and PC7 have been identified as key candidate convertases mediating VEGF-C maturation/activation [49,50]. As furin-like proprotein convertase family members share overlapping substrate specificities, VEGF-C is generally considered to be a common substrate for several convertases, including PCSK6 [51,52]. In addition, the maturation and activation of pro-VEGF-C have been shown to be inhibited by the prosegments, potent inhibitors of proprotein convertases such as PCSK6, which further supports the potential involvement of PCSK6 in the maturation and activation of VEGF-C [50].

#### 2.2.4. Matrix Metalloproteinases (MMPs)

Strong experimental evidence suggests that PCSK6 activates multiple MMPs, including MMP2, MMP9, MMP14 (MT1-MMP), MMP15 (MT2-MMP), MMP24 (MT5-MMP) [53,54,55,56,57]. In smooth muscle α-actin-positive cells, PCSK6 engages in the interaction with MMP14/MMP2 and the genetic ablation of PCSK6 reduced expression of extracellular matrix remodeling enzymes in mice [54]. In PCSK6 overexpressing keratinocytes, MMP14 and MMP15 protein levels were increased, which led to enhanced collagenase activity and consequent collagen degradation [55]. Treatment of U937 and human umbilical vein endothelial cells (HUVECs) with oxysterols and aldehyde 4-hydroxy-2-nonenal—the principal pro-atherogenic molecules derived from oxidized low-density lipoproteins (LDLs)—induced active PCSK6 levels and increased activity of MMP9. More importantly, siRNA mediated downregulation of PCSK6 attenuated oxysterols and HNE-induced activation of MMP9, suggesting a mechanistic association between PCSK6 and MMP9 activity [53].

#### 2.2.5. Adhesion Molecules

Epithelial cadherin (E-cadherin), synthesized as an inactive propeptide precursor in epithelial cells, is a calcium-dependent cell–cell adhesion molecule localized at cell–cell junctions [58]. The proprotein convertases furin, PC7 and PCSK6, have been shown to modulate cell–cell adhesion by processing E-cadherin synergistically [59]. Furin has been identified as cleaving E-cadherin at the cleavage site (RQKR↓) [23]. Moreover, PCSK6 deficiency leads to redistribution and remodeling of E-cadherin at cell–cell junctions, indicating a role for PCSK6 in maintaining junctional integrity [60]. However, E-cadherin undergoes two independent post-translational processing steps and inhibition of PCs did not completely block E-cadherin processing, suggesting the involvement of additional proteolytic mechanisms for the maturation of E-cadherin [61].

#### 2.2.6. Protease-Activated Receptor 1 (PAR-1)

PAR-1 is a G-protein-coupled receptor with seven transmembrane domains located on the cell surface, which can be cleaved at distinct amino acid motifs present in the extracellular domain by different proteases to generate biased signaling responses [62]. Cleavage of the extracellular domain of PAR-1 reveals a tethered ligand that directly binds the activation domain, thereby inducing several intracellular signaling pathways. PAR-1 signaling regulates various physiological and pathological processes, such as inflammation, proliferation, migration, thrombogenesis, angiogenesis, vascular permeability and fibroblast activation [63,64]. Thrombin is the prototypic and best-characterized agonist of PAR-1, cleaving the receptor at the canonical Arg41↓ site within the N-terminal extracellular domain. Interestingly, PCSK6 has been reported to cleave PAR-1 at the non-canonical site Arg46↓, thereby generating a distinct biased signaling profile [65].

#### 2.2.7. Corin

Corin is a trypsin-like serine protease synthesized as an inactive single-chain zymogen [4]. Corin is activated through proteolytic cleavage on the cell surface at a site between the scavenger receptor domain and the protease domain [66]. Corin plays a crucial role in blood pressure regulation and in proper cardiac performance by converting pro-atrial natriuretic peptide (pro-ANP) into its active form, ANP [67,68]. Interestingly, siRNA-mediated knockdown of PCSK6 inhibits Corin activation, whereas overexpression of PCSK6 enhances Corin activation. In line, Corin activation and pro-ANP processing are absent in PCSK6-deficient mice and Corin-dependent pro-ANP processing is also impaired in a hypertension patient carrying a PCSK6 mutation [69].

Soluble Corin and PCSK6 follow distinct secretory pathways within cells, which explains why soluble Corin undergoes PCSK6-dependent activation in conditioned medium experiments, despite the fact that PCSK6 does not activate Corin intracellularly. Notably, activation of Corin does not depend on its transmembrane domain or its pro-peptide region, indicating that membrane association and certain structural elements are dispensable for PCSK6-mediated processing [70]. Overall, it is evident that the propeptide of Corin is an important substrate of PCSK6.

#### 2.2.8. A Disintegrin and Metalloproteinase with Thrombospondin Motif (ADAMTS)-4/Aggrecanase 1 and ADAMTS-5/Aggrecanase 2

ADAMTS-4 and ADAMTS-5 are the principal aggrecanases driving cartilage degeneration in arthritis. ADAMTS-4 and -5 are generated as inactive zymogens and must undergo proteolytic cleavage to become enzymatically active, thereby contributing to cartilage degradation [71,72]. PCSK6 has been shown to activate both recombinant pro-ADAMTS-4 and pro-ADAMTS-5 in vitro [73,74], and to facilitate ADAMTS-4 and -5-dependent aggrecan degradation in models of osteoarthritic cartilage or cytokine-stimulated cartilage [74]. A study with ex vivo joint inflammation models showed that ADAMTS-5 was induced in tenocytes, synovium and in patellar rather than femoral articular cartilage. Furthermore, increased proteoglycan degradation in cartilage has been shown to correlate with increased PCSK6 expression levels, suggesting that post-translational activation of ADAMTS-5 by PCSK6 may represent a key mechanism contributing to arthritic pathology [75]. Taken together, ADAMTS-4 and ADAMTS-5 zymogens have been shown to be substrates of PCSK6 and PCSK6-dependent activation of ADAMTS-4 and ADAMTS-5 may be involved in cartilage degradation.

#### 2.2.9. PRR

The PRR is a cell surface receptor best known for its role in the renin–angiotensin system (RAS), where it enhances the local conversion of angiotensinogen to angiotensin I/II by binding to prorenin/renin, thereby influencing salt–water balance and blood pressure [76]. Beyond its classical RAS-related functions, PRR is now recognized as a multifunctional receptor that is essential for vacuolar H(+)-ATPase (V-ATPase) activity and acts as an intermediary in the Wnt signaling pathway [77]. PRR has been implicated in a range of pathological processes, such as hypertension, renal disease, inflammation, fibrosis, and cardiovascular disorders [78,79]. PCSK6 has been identified as the protease responsible for cleaving PRR at a conserved consensus motif (R-X-X-R↓) in prostate cancer cells, generating soluble PRR (sPRR). This PCSK6-mediated cleavage is required for proper V-ATPase activity and supports the growth of prostate cancer cells, highlighting a functional link between PCSK6, PRR processing, and disease progression [80].

#### 2.2.10. Endothelial Lipase and Lipoprotein Lipase

Endothelial lipase (EL) and lipoprotein lipase (LPL) are triglyceride lipase family members which are essential for high-density lipoprotein (HDL) metabolic regulation. EL primarily hydrolyzes phospholipids in HDL particles, whereas LPL hydrolyzes triglycerides in triglyceride-rich lipoproteins and also contributes to HDL remodeling [81,82]. HDL functions as a key anti-atherogenic lipoprotein, facilitating reverse cholesterol transport through the removal of excess cholesterol from peripheral tissues and subsequent hepatic excretion. Overall, HDL thereby maintains cholesterol homeostasis and reduces the risk of atherosclerotic cardiovascular disease [83,84]. PCSK6 has been shown to cleave the 68 kD full-length EL at the RNKR^330^↓ motif, resulting in a truncated 40 kD N-terminal fragment. Notably, this truncated 40 kD fragment seems inactive as it failed to hydrolyze HDL lipoproteins [85]. Similar to EL cleavage, LPL cleavage by PCSK6 also leads to its inactivation. PCSK6-induced inactivation of LPL can be further enhanced by Angiopoietin-like protein 3 ultimately leading to the inhibition of both the catalytic and noncatalytic functions of LPL [86]. Interestingly, transient PCSK6 overexpression in HEK293 cells induced EL and LPL cleavage, further supporting an important role of PCSK6 in the proteolysis and inactivation of both EL and LPL [87].

**Table 1:** The major (potential) substrate categories of PCSK6 in mammals.

Category	PCSK6 Substrates	Type of Evidence					Function	Potentially Associated with Pulmonary Fibrosis	References
		Direct Biochemical Cleavage by Purified or Recombinant PCSK6	PCSK6-Dependent Processing (Genetic Loss/Gain-of-Function Experiments)	Changes in Substrate Abundance or Activity without Direct Cleavage Evidence	Inferred from Other Proprotein Convertases or Consensus Cleavage Motifs	Cleavage Reported for Other Substrate Family Members			
TGF-β family ligands (propeptide)	TGF-β	No	No	Yes	Yes	Yes	Proteolytic activation of latent precursors, essential for embryogenesis and skeletal development. PCSK6 deficiency leads to left–right inversus and malformations	Yes	[30,31,32,33,36]
	Nodal	No	Yes	No	Yes	Yes			
	BMP	Yes	Yes	–	Yes	Yes			
	GDF15	Yes	Yes	–	Yes	Yes			
Growth factors (propeptide)	IGF-II,	No	Yes	Limited	Yes	Yes	Activation of growth factors, contributing to growth regulation, promoting vascular and tissue remodeling	Yes	[38,39,40,45,46,47]
	PDGF-A/B,	No	Yes	Yes	Yes	Yes			
	VEGF-C	No	No	Yes	Yes	Yes			
Receptors	Propeptide of IRB	No	Yes	Yes	Yes	No	Promoting the maturation of IRB isoform of the insulin receptor	No	[41]
	Propeptide of PAR-1	Yes	Yes	–	Yes	No	Activating PAR-1 through non-canonical cleavage to generate biased downstream signaling; cleaving full-length PRR to generate soluble PRR, a step required for proper V-ATPase activity	Yes	[65,80]
	PRR	Yes	Yes	–	Yes	No		Yes	
Proteinases	Propeptide of MMP2,	Yes	Yes	–	Yes	Yes	Promoting the maturation	Yes	[53,54,55,56,57]
	Propeptide of MMP9	No	Yes	Yes	Yes	Yes			
	Propeptide of MMP14 (MT1-MMP)	Yes	Yes	–	Yes	Yes			
	Propeptide of MMP15 (MT2-MMP)	No	Yes	Yes	Yes	Yes			
	Propeptide of MMP24 (MT5-MMP)	Yes	Yes	–	Yes	Yes			
	Propeptide of ADAMTS-4/5	Yes	Yes	–	Yes	Yes	Removing the pro-domain and activating the aggrecanases which drive cartilage degradation in arthritic disease	No	[73,74,75]
	EL	Yes	Yes	–	Yes	Yes	Cleaving and inactivating EL and LPL	No	[85,86,87]
	LPL	No	Limited	Yes	Yes	Yes			
Adhesion Molecules	Propeptide of E-cadherin	No	Yes	Yes	Yes	No	Processing the precursor of E-cadherin	Yes	[23,59,60,61]
Corin	Propeptide of Corin	Yes	Yes	–	Yes	No	PCSK6 activates Corin, which subsequently converts pro-ANP into its active form—key in regulation of blood pressure and cardiac function	Yes	[69,70]

BMP, mature bone morphogenetic protein; TGF-β, transforming growth factor-β; GDF15, growth differentiation factor 15; IGF-II, insulin-like growth factor-II; PDGF-A/B, platelet-derived growth factor-A/B; VEGF-C, vascular endothelial growth factor-C; IRB, insulin receptor B; PAR-1, protease-activated receptor 1; PRR, prorenin receptor; MMP, matrix metalloproteinase; MT1/2/5-MMP, membrane-type 1/2/5 matrix metalloproteinase; V-ATPase, vacuolar H(+)-ATPase; ADAMTS-4/5, a disintegrin and metalloproteinase with thrombospondin motif-4/5; EL, endothelial lipase; LPL, lipoprotein lipase; E-cadherin, epithelial cadherin; ANP, atrial natriuretic peptide. When evidence showing direct biochemical cleavage by PCSK6 is positive, changes in substrate abundance or activity without direct cleavage evidence are presented using “–” to indicate it is not applicable. PCSK6-cleavage sites in the described (potential) substrates are shown in the Supplementary Table S1, which also indicates the convertases known to cleave the substrates.

## 3. PCSK6 in Disease Pathophysiology

**Figure 2:**
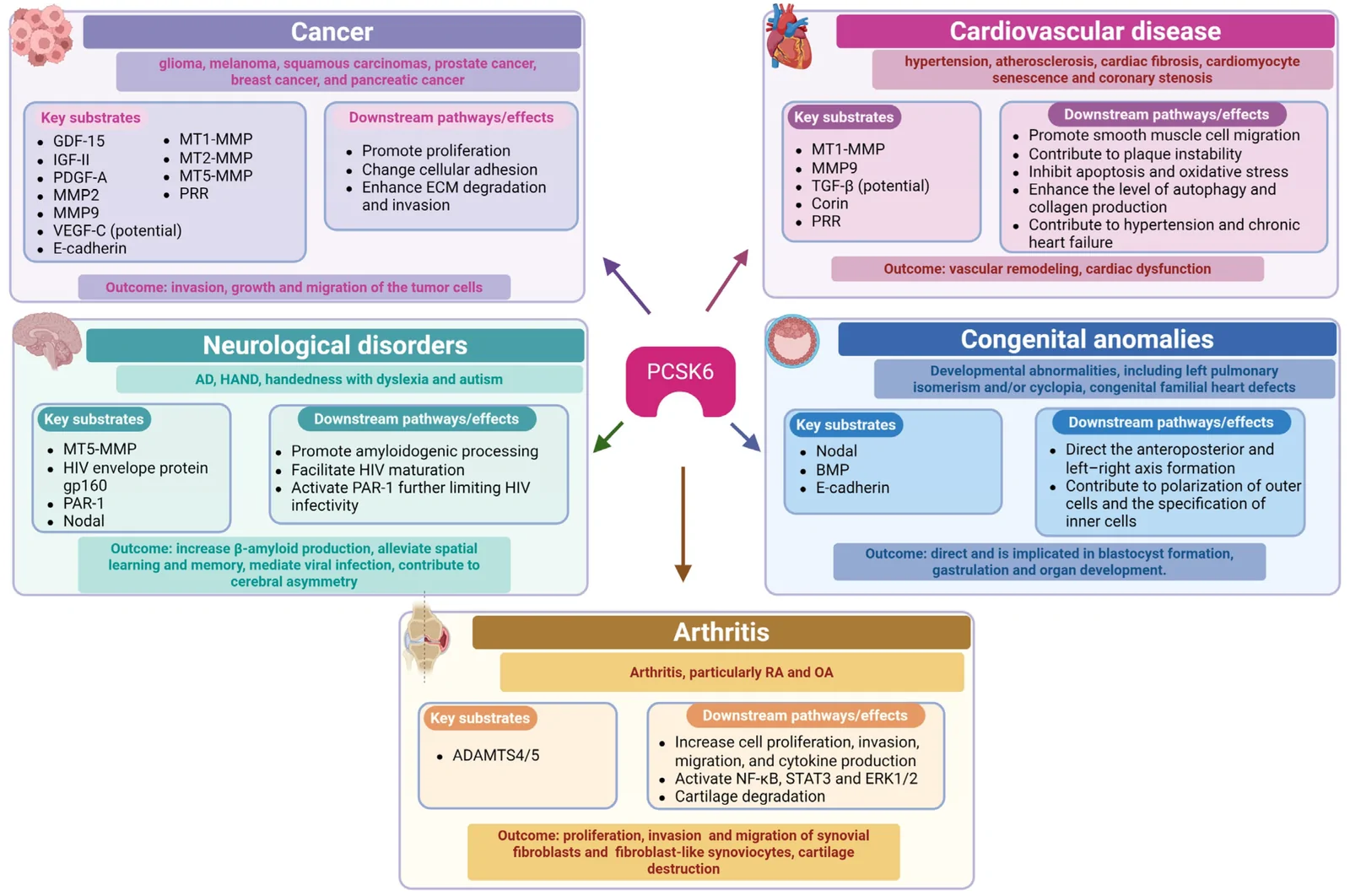
Underlying mechanism of PCSK6 in various diseases. The key substrates potentially activated by PCSK6 and hypothesized downstream pathways or effects in various diseases, including cancer, cardiovascular disease, neurological disorders, congenital anomalies and arthritis, are shown in this figure. GDF15, growth differentiation factor 15; IGF-II, insulin-like growth factor-II; PDGF-A, platelet-derived growth factor-A; VEGF-C, vascular endothelial growth factor-C; MMP, matrix metalloproteinases; MT-MMP, membrane-type matrix metalloproteinase; PRR, prorenin receptor; ECM, extracellular matrix; MT1/5-MMP, membrane-type 1/15 matrix metalloproteinase; TGF-β, transforming growth factor-β; AD, Alzheimer’s disease; HIV, human immunodeficiency virus; HAND, HIV-associated neurocognitive disorder; PAR-1, protease-activated receptor 1; BMP, mature bone morphogenetic protein.

Accumulating evidence indicates that PCSK6 is involved in the pathophysiology of various diseases, including tumor biology, cardiovascular disease, neurological disorders, congenital anomalies and arthritis. In addition, the underlying molecular mechanisms affected by PCSK6 have been increasingly investigated in the past decades. Table 2 provides an overview of the diseases that are associated with PCSK6, and in Figure 2 an overview of the underlying pathophysiological mechanisms is shown (Figure 2).

### 3.1. Tumors

PCSK6 has been implicated in a wide range of tumor types, including glioma, melanoma, squamous carcinomas, prostate cancer, breast cancer, and pancreatic cancer. Owing to its substrate range, PCSK6 occupies a central position at the intersection of growth factors, growth factor receptors, adhesion molecules, angiogenic factors and matrix-remodeling pathways, all of which are relevant to tumor biology including invasion, growth and migration of the tumor cells. PCSK6 overexpression has been observed in cell lines derived from skin tumors. In addition, squamous cell carcinomas obtained from PCSK6 transgenic mice showed deeper invasion and accelerated growth compared to tumors from wild type mice following chemical carcinogenesis [88]. Molecular profiling of primary glioma tissue identified PCSK6 as an invasion associated candidate gene and PCSK6 deficiency suppressed glioma cell invasion in vitro [89]. In prostate cancer, PACE4-altCT, an oncogenic C-terminally modified isoform of PCSK6 containing a novel 30-amino-acid C-terminal sequence with 2 cysteines instead of the conventional 32-amino-acid sequence with 4 cysteines, more efficient autocatalytic activation, and reduced secretion, has been identified as an isoform capable of processing pro-GDF15 and has been proposed as both a potential biomarker and a pharmacological target [9,10].

In terms of mechanism, PCSK6 appears to enhance tumor cell invasion and migration through processing substrates that disrupt the normal basement membrane architecture. This may include direct effects on E-cadherin redistribution and cytoskeleton arrangement, as well as activation of MMP2, MMP9, MT1-MMP, MT2-MMP and MT5-MMP, which degrade collagen and gelatine [55,60,90]. PCSK6 may also promote tumor cell proliferation and angiogenesis, likely through promoting cell-cycle progression, activation of IGF-II, PDGF-A and VEGF-C, and cleavage of PRR [39,45,50,60,80]. In breast cancer, PCSK6 expression negatively correlates with multiple components of the immune microenvironment, including immune activating molecules, chemokines, chemokine receptors, and immunosuppressive factors [91]. In estrogen receptor-positive breast cancer and skin tumors, PCSK6 silencing or inhibition reduced proliferation and increased cellular quiescence highlighting PCSK6 as a promising therapeutic target for those tumor types [92,93].

### 3.2. Cardiovascular Disease

Multiple studies have shown a strong association between PCSK6 and diverse cardiovascular pathophysiologies, including hypertension, vascular remodeling in atherosclerosis, cardiac fibrosis, cardiomyocyte senescence and coronary stenosis [94]. In atherosclerotic disease, however, the role of PCSK6 remains somewhat controversial. For example, mice transplanted with bone marrow from *PCSK6*-deficient mice developed higher plaque burden, but exhibited increased plaque stability compared to mice transplanted with control (wildtype) bone marrow [95]. In a prospective cohort study, individuals carrying the PCSK6 rs1531817 polymorphism, a variant associated with decreased PCSK6 activity, displayed milder coronary stenosis and improved prognosis following premature myocardial infarction [96]. In contrast, the same PCSK6 variant was associated with progression of maximum internal carotid intima-media thickness in high-risk cardiovascular patients, as well as increased plaque smooth muscle α-actin-positive cell content in carotid arteries, both signs of more severe disease [54]. Transcriptomic and tissue microarray profiling further revealed increased PCSK6 expression in the fibrous cap of unstable carotid atherosclerosis, correlated with smooth muscle cell activity, inflammation and extracellular remodeling [97]. Mechanistically, PCSK6 promoted smooth muscle cell migration in response to PDGF-B via MT1-MMP activation and contributed to plaque instability via MMP9 activation [53,54]. Although PCSK6 was associated with a reduced plaque burden, the overall evidence suggests that PCSK6 contributes to disease development and progression.

Beyond atherosclerotic disease, PCSK6 is also implicated in several other cardiovascular diseases through distinct mechanisms. In doxorubicin-induced cardiac injury, PCSK6 overexpression inhibited apoptosis and oxidative stress, protected cardiac function through Sirtuin 1/Forkhead box O3a (SIRT1/FOXO3a) and enhanced the level of autophagy [98]. Following myocardial infarction, PCSK6 expression was found to be elevated in human serum and hearts of mice. Notably, PCSK6 deficiency in cardiomyocytes disrupted TGF-β activation and mothers against decapentaplegic homolog 3 (SMAD3) translocation in fibroblasts. Moreover, PCSK6 overexpression increased collagen production and promoted cardiac fibrosis in mice [36].

PCSK6 also seems to play a role in hypertension. Indeed, PCSK6-deficient mice develop hypertension and a PCSK6 mutation in the catalytic domain was associated with a deficiency in Corin activation in a hypertension patient [69], suggesting that PCSK6-dependent Corin activation may protect against hypertension. In addition to Corin activation, the effect of PCSK6 on hypertension may also depend on sPRR, as PCSK6 has been found to cleave full-length PRR to generate sPRR [80]. We hypothesize that, given the role of sPRR in hypertension and chronic heart failure [99], PCSK6 may promote hypertension, at least in part, through increased sPRR production.

### 3.3. Neurological Disorders

Several neurological conditions have been linked to PCSK6 activity, including Alzheimer’s disease (AD), human immunodeficiency virus (HIV)-associated neurocognitive disorder (HAND), handedness, dyslexia and autism. In brain tissues from AD patients and AD mouse models, PCSK6 expression levels were increased and these increased levels were associated with the pathogenesis of AD. In N2A^APP^ cells, neuroblastoma cells bearing a genetic mutation involved in the development of AD, genetic deficiency of PCSK6 suppressed the activation of MT5-MMP, thereby reducing amyloidogenic processing of amyloid precursor protein. Cleavage and activation of MT5-MMP were taking place at the RRRNKR motif in the N-terminal propeptide domain. Importantly, in a mouse model for AD, PCSK6 deficiency reduced β-amyloid production and alleviated hippocampal long-term potentiation (LTP) and long-term spatial learning and memory [56].

PCSK6 is also involved in Familial British dementia (FBD). The disease-causing protein BRI-L, generated by a mutation in the integral membrane protein 2B (BRI/ITM2B) protein, is the precursor of the Abri peptide, a component of amyloid deposits in the brain. Although the evidence is not strong, both wild-type BRI and the mutant counterpart BRI-L were shown to be processed by PCSK6. However, only the amyloidogenic peptide derived from BRI-L is pathogenic leading to FBD [100].

Beyond neurodegeneration, PCSK6 may also contribute to viral pathogenicity specifically through cleavage of viral peptides. Indeed, cleavage of the HIV envelope protein gp160 into gp120 and gp41, needed for viral membrane fusion, is mediated by furin at the R-X-K/R-R↓ motif [101] and may also be mediated by PCSK6 [102]. Conversely, PCSK6-dependent PAR-1 activation can initiate a negative feedback loop that suppresses PCSK6 activity and limits HIV infectivity in the brain, thereby reducing HIV-induced neuroinflammation and potentially mitigating the development of HAND [65].

Moreover, PCSK6 plays a role in the mechanisms underlying cerebral asymmetry. By cleaving Nodal, a key determining protein of left–right axis formation, PCSK6 is associated with handedness phenotypes, including those observed in individuals with dyslexia [103,104]. Additionally, PCSK6 expression correlates with dimensional schizotypy and autism-related traits, suggesting broader roles in neurodevelopmental variation [105].

### 3.4. Congenital Anomalies and Developmental Disorders

During early embryonic development, PCSK6 plays a pivotal role by activating the TGF-β family members, such as Nodal, Lefty and BMPs, which are important for directing the anteroposterior (A/P) and left–right (L/R) axis formation [106]. PCSK6-deficient embryos exhibited severe developmental abnormalities, including left pulmonary isomerism and/or cyclopia [30]. PCSK6 has also been shown to be essential for blastocyst formation. Indeed, PCSK6 contributed to the polarization of outer cells and the specification of inner cells through regulation of E-cadherin. In *Furin* and *PC7* double-deficient blastocysts, PCSK6 was sufficient to rescue compaction, although this occurred with a delay, which unmasked overlapping and complementary functions among these three PCs. In contrast, in triple deficient blastocytes that lack Furin, PC7, and PCSK6, cell adhesion was largely impaired due to immaturity of E-cadherin and consequent intracellular retention [59].

During gastrulation, Nodal signaling induced PCSK6 expression in the extraembryonic ectoderm, where PCSK6 subsequently activated Nodal, forming a positive regulatory loop that prevents premature differentiation of trophoblast stem cells [107]. Notably, genome-wide linkage analysis in families with congenital heart defects identified PCSK6 as one of four candidate genes underlying the disease, further underscoring its importance in early developmental processes [108].

### 3.5. Arthritis

Several studies have shown a strong association between PCSK6 and arthritis, particularly rheumatoid arthritis (RA). PCSK6 was upregulated in the synovial tissues of RA patients. PCSK6 deficiency in fibroblast-like synoviocytes from rats with collagen-induced arthritis, as well as in synovial fibroblasts from RA patients, resulted in reduced cell proliferation, invasion, migration, and cell-cycle progression as well as inflammatory cytokine production. These findings suggest that inhibition of PCSK6 may exert protective effects in RA [38,109]. Interestingly, the detrimental effect of PCSK6 appeared to be mediated through activation of the nuclear factor (NF)-κB, signal transducer and activator of transcription 3 (STAT3) and extracellular signal-regulated kinase (ERK)1/2 signaling pathways [110]. Additionally, circular RNA circ_0002984 induced PCSK6 production via binding miR-543, thereby promoting the pathogenic phenotypes of RA fibroblast-like synoviocytes, indicating a regulatory axis involving non-coding RNA in RA [111].

PCSK6 has also been implicated in playing a detrimental role in osteoarthritis (OA). A PCSK6 variant, i.e., the minor allele at rs900414, was more frequently found in asymptomatic compared to symptomatic knee OA, suggesting PCSK6 may exacerbate the onset and progression of OA symptoms. Consistently, PCSK6-deficient mice show reduced pain sensitivity in OA models, supporting a role for PCSK6 in OA-related nociception [112]. Given that ADAMTS4 and ADAMTS5 are implicated in cartilage destruction in arthritis and that PCSK6 activates ADAMTS4 and ADAMTS5, PCSK6 may likely contribute to the pathophysiology of arthritis through ADAMTS4 and ADAMTS5 activation [75].

Overall, PCSK6 appears to exert detrimental effects in both RA and OA, positioning it as a promising therapeutic target in arthritis.

**Table 2:** The evidence overview of PCSK6 involvement in disease pathophysiology.

Disease		Evidence Type				Direction of Effect	References
		Genetic Associations	Expression Correlations	In Vitro Functional Data	In Vivo Causal Evidence		
Tumors (including glioma, melanoma, squamous carcinomas, prostate cancer, breast cancer, pancreatic cancer).		PCSK6 as an invasion associated candidate gene.	1. PCSK6 overexpression has been observed in cell lines derived from skin tumors; 2. Negatively correlates with multiple components of the immune microenvironment.	1. Direct effects on E-cadherin redistribution and cytoskeleton arrangement, activation of MMP2, MMP9, MT1-MMP, MT2-MMP, MT5-MMP; 2. Promoting tumor cell proliferation. 3. PCSK6 deficiency suppressed glioma cell invasion in vitro.	Squamous cell carcinomas obtained from PCSK6 transgenic mice showed deeper invasion and accelerated growth.	Detrimental	[39,45,50,55,60,80,88,89,90,91,92,93]
Cardiovascular disease	Atherosclerosis	1. Individuals carrying the PCSK6 rs1531817 polymorphism, displayed milder coronary stenosis and improved prognosis following premature myocardial infarction; 2. Whereas the same variant is associated with progression of more severe disease.	Increased PCSK6 expression in the fibrous cap of unstable carotid atherosclerosis, correlated with smooth muscle cell activity, inflammation and extracellular remodeling.	PCSK6 promoted smooth muscle cells migration in response to PDGF-B via MMP14 activation and contributed to plaque instability by MMP9 activation.	Mice transplanted with bone marrow from PCSK6 deficient−mice developed higher plaque burden, but exhibited increased plaque stability.	Dual	[36,53,54,69,80,94,95,96,97,98]
	Cardiac injury, myocardial infarction and fibrosis		Following myocardial infarction, PCSK6 expression is elevated in human serum and hearts of mice.	1. In cardiac injury, PCSK6 protected cardiac function through SIRT1/FOXO3a. 2. PCSK6 deficiency in cardiomyocytes disrupted TGF-β activation and SMAD3 translocation in fibroblasts.	1. In cardiac injury, PCSK6 overexpression inhibited apoptosis and oxidative stress, enhanced the level of autophagy; 2. PCSK6 overexpression increased collagen production and promoted cardiac fibrosis in mice.	Dual	
	Hypertension	A PCSK6 mutation in the catalytic domain was associated with a deficiency in Corin activation.			PCSK6 deficient mice develop hypertension.	Protective	
Neurological disorders	Alzheimer’s disease (AD)		In brain tissues from AD patients and AD mouse models, PCSK6 increased levels were associated with the pathogenesis of AD.	Genetic deficiency of PCSK6 suppressed the activation of MT5-MMP, thereby reducing amyloidogenic processing of amyloid precursor protein.	In a mouse model for AD, PCSK6 deficiency reduced β-amyloid production and alleviated hippocampal LTP and long-term spatial learning and memory.	Detrimental	[56,65,100,101,102,103,104,105]
	HIV-associated neurocognitive disorder (HAND)		In postmortem brains of individuals exhibiting HAND, the mRNA level of PCSK6 is significantly increased.	1. cleavage of the HIV envelope protein gp160 into gp120 and gp41, which are needed for viral membrane fusion; 2. PCSK6 cleave PAR-1 at Arg46↓, which inhibits HIV infectivity.		Dual	
	Handedness with dyslexia, autism	Genome-wide association studies reported an association between PCSK6 polymorphisms and handedness with dyslexia or autism.				Unidentified	
Congenital anomalies and development disorders		Genome-wide linkage analysis in families with congenital heart defects identified PCSK6 as one of four candidate genes.		1. In Furin and PC7 double deficient blastocysts, PCSK6 was sufficient to rescue compaction, but in triple deficient blastocytes, cell adhesion was largely impaired due to lack of mature E-cadherin; 2. Activating Nodal during gastrulation to prevent precocious differentiation of trophoblast stem cells.	1. Activating the TGF-β family members, such as Nodal, Lefty and BMPs, directing the anteroposterior and left–right axis formation; 2. PCSK6 deficiency leads to situs ambiguous combined with left pulmonary isomerism and/or cyclopia.	Beneficial	[30,59,106,107,108]
Arthritis	Rheumatoid arthritis (RA)		PCSK6 was upregulated in the synovial tissues of RA patients.	1. PCSK6 deficiency decreased cell proliferation, invasion, migration, cell-cycle progression and inflammatory cytokine release in fibroblast-like synoviocytes and synovial fibroblasts; 2. The detrimental effect of PCSK6 was mediated through activation of NF-κB, STAT3 and ERK1/2 pathway; 3. Circular RNA circ_0002984 upregulated PCSK6 production by binding miR-543, promoting RA fibroblast-like synoviocytes phenotypes.		Detrimental	[38,75,109,110,111,112]
	Osteoarthritis (OA)	A PCSK6 variant rs900414, was more frequently found in asymptomatic knee OA.		PCSK6 presents the primary activity in OA cartilage, and PCSK6 suppression inhibit aggrecanase-induced aggrecan degradation.	PCSK6 deficient OA mice show protective effect against pain.	Detrimental	

MMP, matrix metalloproteinases; PDGF-B, platelet-derived growth factor-B; SIRT1/FOXO3a, Sirtuin 1/Forkhead box O3a; TGF-β, transforming growth factor-β; SMAD3, mothers against decapentaplegic homolog 3; HIV, human immunodeficiency virus; PC7, proprotein convertase 7; LTP, long-term potentiation; MT5-MMP, membrane-type 5 matrix metalloproteinase; BMP, mature bone morphogenetic protein; NF-κB, nuclear factor-κB; STAT3, signal transducer and activator of transcription 3; ERK1/2, extracellular signal-regulated kinase 1/2.

## 4. Potential Role of PCSK6 in Pulmonary Fibrosis

**Figure 3:**
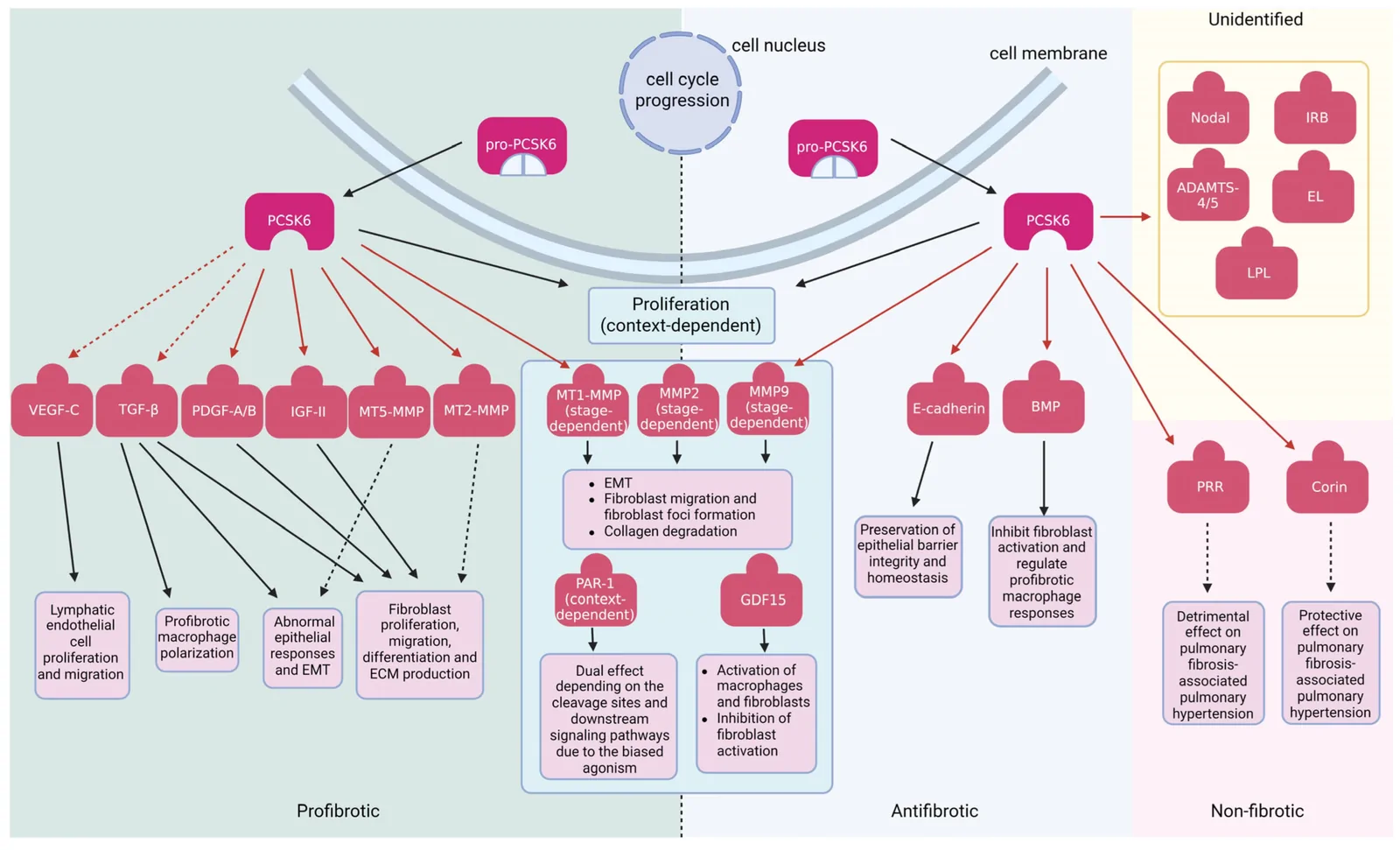
Proposed potential roles of PCSK6 in pulmonary fibrosis. Shown are the (potential) PCSK6 substrates that may exert pro-, anti- or non-fibrotic processes in pulmonary fibrosis. Whether these potential substrates are indeed cleaved by PCSK6 and whether the proposed processes are involved in pulmonary fibrosis remains hypothetical and needs further investigation. Red solid arrows indicate substrate activation by PCSK6 with direct evidence. Substrate activation by PCSK6 inferred from indirect evidence is indicated with a red dashed arrow. Notably, enzyme-substrate co-localization and accessibility remain unknown. We hypothesize that PCSK6 may contribute to pro-fibrotic signaling by promoting the maturation and activation of multiple substrates, including TGF-β, IGF-II, VEGF-C, and PDGF-A/B by proteolytic cleavage of their preproteins. PCSK6 may further exacerbate fibrotic remodeling by facilitating the activation of MMP2, MMP9, MT-1-MMP, MT2-MMP, MT5-MMP and PAR-1, although MT-1-MMP, MMP2, MMP9 and PAR-1 may also exert anti-fibrotic effects depending on the context and phases of remodeling. For example, by enhancing cellular proliferation, PCSK6 may either exacerbate or attenuate fibrosis depending on the cell type involved. Moreover, PCSK6 may mitigate fibrotic progression by processing the E-cadherin precursor, thereby supporting epithelial integrity. In addition, PCSK6 may inhibit fibroblast-to-myofibroblast differentiation, extracellular matrix production, and reduce the pro-fibrotic macrophage responses by activating BMP. GDF15 may play a dual role in pulmonary fibrosis as described. PCSK6 may play a non-fibrotic role in ILD by maintaining a normal blood pressure via activation of Corin or a detrimental effect by promoting hypertension by increasing sPRR levels. Downstream effects induced by these substrates with evidences in the lung are indicated with black solid arrows. Hypothetical downstream effects without evidence in the lung, but inferred from pathophysiology in other organs, are represented with black dashed arrows.

As a member of the furin-like proprotein convertase family, PCSK6 possesses proteolytic cleavage potential that enables it to activate a wide range of precursor proteins. This broad substrate repertoire positions PCSK6 as a central regulator of numerous physiological and pathological processes as outlined above. Landmark studies by Oldham et al. [5] and Berger et al. [6] reported that PCSK6 levels were increased in peripheral blood and lung tissue of IPF patients. Immunohistochemical staining in the study of Oldham et al. [5] showed increased cytoplasmic PCSK6 levels in ciliated epithelial cells and alveolar epithelial cells in IPF lung tissue compared to normal lung control sections. On the contrary, Berger et al. [6] identified enhanced PCSK6 expression in lymphatic endothelial cells and HAS1+ fibroblasts in IPF compared to control and other fibrotic ILD subtypes using publicly available single-cell RNA-sequencing datasets. The apparent discrepancy in the cell types identified as PCSK6-expressing warrants further investigation, which may be of importance to identify the role of PCSK6 in the pathophysiology of IPF, and raises the question of whether PCSK6 has cell-type-specific effects in IPF on fibrogenesis, other disease-specific pathways, or whether it is just a prognostic biomarker for IPF. Since PCSK6 was not associated with respiratory-specific outcomes in IPF and overall survival in mixed fibrotic ILD [5,6], a direct causal link between plasma PCSK6 levels and pulmonary fibrosis cannot be inferred. This notion is further strengthened by the observation that PCSK6 was significantly associated with a lower hazard of 10% FVC decline in the non-IPF subgroup [6]. However, drawing on insights from other disease contexts, most of which involve the downstream effects of PCSK6-mediated substrate activation, we propose several potential untested mechanisms, as outlined in detail below, through which PCSK6 may contribute to pulmonary fibrosis (Figure 3).

### 4.1. Potential Pro-Fibrotic Role of PCSK6

TGF-β, IGF-II and PDGF are well-established drivers of myofibroblast differentiation and pro-fibrotic remodeling, and they represent major modulators in the development and progression of pulmonary fibrosis [113,114,115]. As PCSK6 has been shown to promote the maturation of IGF-II and PDGF-A/B, and may potentially activate TGF-β through proteolytic cleavage of their latent precursor forms, PCSK6 may promote fibrotic responses through activation of these proteins. In cardiomyocytes, PCSK6 deficiency decreased collagen production by impairing TGF-β activation and downstream SMAD3 signaling, whereas cardiomyocyte-specific overexpression of PCSK6 in mice enhanced collagen expression and aggravated cardiac fibrosis [36]. These findings provide mechanistic insight into how PCSK6-mediated TGF-β activation may contribute to pro-fibrotic processes. In addition, PDGF signaling also plays a critical role in the initiation and progression of fibrotic diseases [116]. Given that PCSK6 facilitates PDGF-A/B and IGF-II maturation, it is plausible to predict that PCSK6 enhances PDGF- or IGF-II-dependent pro-fibrotic signaling in the lung, although this remains to be experimentally validated.

Recent studies have highlighted lymphangiogenesis as a key process in the progression of pulmonary fibrosis [117]. VEGF-C is a central driver of pathological lymphatic expansion in this context [118]. PCSK6 may further exacerbate fibrotic remodeling by modulating VEGF-C–dependent lymphangiogenic pathways, thereby facilitating the pathophysiology of pulmonary fibrosis [119,120]. But this hypothesis remains untested.

### 4.2. Both Pro-Fibrotic and Anti-Fibrotic Roles of PCSK6

Several studies have illustrated that PCSK6 has been reported to activate several MMPs, including MMP2, MMP9, MT1-MMP, MT2-MMP and MT5-MMP. MMPs are well known for their ability to degrade extracellular matrix components including collagen. Importantly, they function not only as anti-fibrotic enzymes, but also as active drivers of fibrotic remodeling [121]. For example, the gelatinases MMP2 and MMP9 can promote epithelial–mesenchymal transition, facilitate fibroblast migration through basement membranes and contribute to fibroblast foci formation and activate latent TGF-β1, thereby enhancing fibrosis progression [121,122]. On the other hand, MMP2 and MMP9 have been shown to degrade collagen and collagen fragments [123,124], which is important for extracellular matrix remodeling and resolution of pulmonary fibrosis [125]. In this regard, PCSK6 may present anti-fibrotic effect through promoting extracellular matrix remodeling. MT1-MMP, which is capable of activating pro-MMP2 and latent TGF-β as well as degrading excess collagen, may exert either pro-fibrotic or anti-fibrotic effect during different phases [126,127,128]. The contribution of MT2-MMP to fibrosis remains incompletely defined, although current evidence suggests that it may facilitate fibrogenesis primarily by enhancing fibroblast migration, invasiveness, and extracellular matrix remodeling [129,130,131]. MT5-MMP, best characterized in neurological disorders and predominantly expressed in the brain and epithelial cells, may indirectly promote fibrotic responses by exacerbating epithelial injury and driving epithelial–mesenchymal transition through activation of pro-MMP2 [132,133]. Overall, activation of these MMPs by PCSK6 may exert both pro- and anti-fibrotic responses.

PAR-1, expressed on multiple cell types relevant in fibrotic disease, has been implicated in pulmonary fibrosis [134]. PAR-1 can be cleaved at different cleavage sites, thereby activating distinct signaling pathways, referred to as biased agonism, that may exert opposing roles in pulmonary fibrosis. PAR-1 activation by PAR-1 agonist peptide induced pro-fibrotic responses in fibroblasts and pharmacological PAR-1 inhibition with P1pal-12 attenuated the fibrotic response induced by bleomycin [135]. However, activated protein C, which can also activate PAR-1, has been shown to limit fibrosis by competing with pro-fibrotic protease activation of PAR-1 or preferentially engaging anti-fibrotic downstream signaling pathways [134,136,137]. As PCSK6 is one of the proteases capable of activating PAR-1, it is reasonable to hypothesize that PCSK6 may further promote or attenuate development of pulmonary fibrosis via PAR-1 dependent pathways. At this moment, however, the role of PCSK6-dependent activation of PAR-1 in pulmonary fibrosis remains elusive.

PCSK6 has been reported to enhance cellular proliferation also in tumor cells, mediated by promoting cell-cycle progression or by activating growth factors [39,60]. The fibrotic consequences of increased proliferation are highly cell-type dependent. Proliferation of fibroblasts or myofibroblasts would be expected to exacerbate fibrotic remodeling. A recent study showed that PCSK6 enhanced proliferation of A549 epithelial cells [138]. Of note, A549 cells are transformed lung adenocarcinoma-derived cells and increased proliferation in such a cell line does not, by itself, establish a pro-fibrotic response in normal alveolar epithelial cells. Despite this limitation this finding raises the possibility of a pro-fibrotic role of PCSK6 as uncontrolled cancerous or aberrant epithelial proliferation and hyperplasia appear to promote a pro-fibrotic environment [139]. Repeated and chronic microinjuries to the lung epithelium, specifically hyperplastic or senescent alveolar epithelial cells, contribute to dysregulated repair and fibroblast activation [140,141]. In contrast, proliferation of normal epithelial cells—particularly in the context of injury repair—is generally considered reparative rather than pro-fibrotic [142]. But the effect of PCSK6 on proliferation of aberrant and normal epithelial cells still needs to be verified.

Additionally, GDF15, a biomarker associated with the severity and prognosis of pulmonary fibrosis, that is activated by PCSK6 cleavage, is thought to play a dual role (pro- and anti-fibrotic effects) in pulmonary fibrosis [143,144]. Indeed, on the one hand, GDF15 promotes fibroblast proliferation and collagen deposition in IPF [143], but may on the other hand also inhibit fibroblast proliferation and activation [144] probably depending on the biological context or stage of fibrosis. Therefore the role of PCSK6-induced maturation of GDF15 in the context of pulmonary fibrosis remains speculative.

### 4.3. Potential Anti-Fibrotic Role of PCSK6

Considering its broad substrate specificity, PCSK6 may also play an anti-fibrotic role in pulmonary fibrosis. Although no direct evidence currently demonstrates an anti-fibrotic function for PCSK6 in the development of pulmonary fibrosis, conditioned medium derived from PCSK6 overexpressing A549 epithelial cells has been shown to decrease collagen deposition in normal human lung fibroblasts potentially via MMP activation, suggesting a potential anti-fibrotic effect of PCSK6 in pulmonary fibrosis [138]. Whether PCSK6 directly acts on fibroblasts or exerts its effect due to changes in other secreted factors or protease activity remains to be established however. BMP has been shown to inhibit fibroblast-to-myofibroblast differentiation, extracellular matrix production, and reduce the pro-fibrotic macrophage responses in recent studies [145,146]. Considering direct evidence showing that PCSK6 is able to activate BMP, it is hypothesized that PCSK6 may inhibit pulmonary fibrosis through activation of BMP. In addition, PCSK6 is capable to activate the precursor of E-cadherin [59,60], which is predominantly expressed by normal epithelial cells where it mediates cell–cell adhesion and maintains epithelial barrier integrity [147], supporting epithelial architecture important for normal lung function. Because epithelial dysfunction is a central feature of fibrotic lung disease, and loss of E-cadherin is associated with the injured lung epithelium in pulmonary fibrosis [148,149]. PCSK6-mediated activation of E-cadherin could theoretically help preserve epithelial barrier integrity and epithelial homeostasis, thereby potentially suppressing the progression of pulmonary fibrosis.

### 4.4. Non-Fibrotic Roles Associated with Pulmonary Fibrosis

Interestingly, endothelial dysfunction and vascular remodeling contribute to the pathogenesis and progression of pulmonary fibrosis, particularly in the development of pulmonary fibrosis-associated pulmonary hypertension [150,151]. PCSK6 has been shown to play a protective role in cardiovascular hypertension by maintaining a normal blood pressure through activation of Corin [69], but may also have detrimental effects by cleaving PRR to generate sPRR [80], which may contribute to hypertension [99]. These studies suggest that PCSK6, through activation of Corin and cleavage of PRR, may play a dual role in non-fibrotic processes, including the development of pulmonary fibrosis-associated pulmonary hypertension, which in turn may affect the severity of pulmonary fibrotic disease and all-cause mortality of pulmonary fibrosis patients.

### 4.5. Experimental Framework for Investigating the Mechanism of PCSK6 in Pulmonary Fibrosis

In order to identify the pathophysiological role of PCSK6 in pulmonary fibrosis, experimental validation of the potential mechanisms proposed is essential. To this end, several experimental approaches could be used, including cell-type-specific PCSK6 deletion or overexpression experiments; rescue experiments with catalytically inactive versus active PCSK6 or substrate specific interventions; direct measurements of substrate cleavage products; activity-based protease assays; spatial assessment of enzyme-substrate colocalization; and comparison with other proprotein convertases like furin, PCSK5 or PCSK7. These approaches could be investigated using complementary experimental models of pulmonary fibrosis like primary fibrotic human lung cells from healthy and fibrotic tissues, precision-cut lung slices from normal and fibrotic human lung tissue exposed to defined pro-fibrotic stimuli, and the murine bleomycin-induced pulmonary fibrosis model. Using these models in combination would help establish whether PCSK6 contributes to fibrotic processes, identify the relevant cell types and substrates, and distinguish PCSK6-specific effects from those mediated by other proprotein convertases.

## 5. Conclusions

In this review, we summarized the molecular biology of PCSK6, including its structural features and mode of activation, substrate specificities, and diverse pathophysiological roles across multiple diseases. Moreover, we proposed several hypotheses regarding the potential involvement of PCSK6 in the development and progression of pulmonary fibrosis. These hypotheses, based upon established functions of PCSK6 in other pathological contexts, support testable hypotheses regarding the potential role of PCSK6 in pulmonary fibrosis and may guide future investigations aiming to define the precise contribution of PCSK6 to pulmonary fibrosis. Notably, most of the hypotheses regarding the effect of PCSK6 on pulmonary fibrosis are based on the mechanisms and roles of PCSK6 in other diseases. Currently, there is only limited evidence that PCSK6 exerts a key role in pulmonary fibrosis, and stronger experimental validation is therefore eagerly awaited. These studies should elucidate whether the role of PCSK6 extends beyond that of a prognostic biomarker for IPF and whether it actively mediates fibrotic remodeling.

## 6. Literature Search Strategy

The PubMed (https://pubmed.ncbi.nlm.nih.gov) database was used to retrieve relevant articles on potential PCSK6 substrates and its role in pathophysiology. The search terms for identification of potential PCSK6 substrates were “PCSK6”, “PACE4”, “Furin convertase”, “furin”, and “Proprotein convertase subtilisin/kexin type 6”. The final search date was 9 July 2026. Criteria to identify PCSK6 substrates were as follows: (1) direct biochemical cleavage by purified or recombinant PCSK6; (2) PCSK6-dependent processing (genetic loss/gain-of-function experiments); (3) changes in substrate abundance or activity without direct cleavage evidence; (4) inferred from other proprotein convertases or consensus cleavage motifs; (5) reported for other substrate family members. The literature used to identify the role of PCSK6 in disease pathophysiology was selected by searching for articles describing a role for PCSK6 or its substrates in any pathophysiological condition.

## Data Availability

The original contributions presented in this study are included in the article. Further inquiries can be directed to the corresponding authors.

## Supplementary Materials

The following supporting information can be downloaded at https://www.mdpi.com/article/10.3390/molecules31213931/s1: Table S1: Overview of major (potential) substrates of PCSK6, cleavage sites, evidence level of PCSK6 processing of the substrate, and references. References [152,153,154,155,156,157,158,159,160] are cited in the supplementary materials.

## Abbreviations

The following abbreviations are used in this manuscript:

PCSK	Proprotein convertase subtilisin/kexin type
IPF	Idiopathic pulmonary fibrosis
SPC4	Subtilisin-like proprotein convertase 4
PACE4	Paired basic amino acid–cleaving enzyme 4
ECM	Extracellular matrix
HAS1	Hyaluronan synthase 1
CRD	Cys-rich domain
ER	Endoplasmic reticulum
HSPG	Heparan sulfate proteoglycan
PC	Proprotein convertase
TGF-β	Transforming growth factor-β
BMP	Bone morphogenetic protein
GDF15	Growth differentiation factor 15
IGF-II	Insulin-like growth factor-II
IRB	Insulin receptor B
SPC	Subtilisin-like proprotein convertase
MLC	Myosin light chain
PDGF-A/B	Platelet-derived growth factor-A/B
VEGF	Vascular endothelial growth factor
MMP	Matrix metalloproteinase
E-cadherin	Epithelial cadherin
HNE	4-hydroxy-2-nonenal
LDL	Low-density lipoproteins
PAR-1	Protease-activated receptor 1
ANP	Atrial natriuretic peptide
ADAMTS	A disintegrin and metalloproteinase with thrombospondin motif
EL	Endothelial lipase
LPL	Lipoprotein lipase
PRR	Prorenin receptor
RAS	Renin–angiotensin system
V-ATPase	Vacuolar H(+)-ATPase
SIRT1	Sirtuin 1
FOXO3a	Forkhead box O3a
SMAD3	Mothers against decapentaplegic homolog 3
AD	Alzheimer’s disease
HAND	HIV-associated neurocognitive disorder
LTP	Long-term potentiation
FBD	Familial British dementia
RA	Rheumatoid arthritis
STAT3	Signal transducer and activator of transcription 3
ERK1/2	Extracellular signal-regulated kinase 1/2
OA	Osteoarthritis
NF-κB	Nuclear factor-κB
