# Supplementary material for: PCSK6 in Pathophysiology and Its Potential Involvement in Pulmonary Fibrosis
Source: Molecules. 2026 Nov 1;31(21):3931. doi: 10.3390/molecules31213931 (PMC13765174; doi:10.3390/molecules31213931)
Supplement: Supplementary file 1 [file molecules-31-03931-s001.zip › molecules-4474504-supplementary.pdf]

Supplementary table S1. Overview of major (potential) substrates of PCSK6, cleavage sites, evidence level of PCSK6 processing of the substrate, and references.

Substrates of PCSK6	Cleavage site or motif for PCSK6	Experimental system	Convertases known to cleave the substrate	References
Propeptide of TGF-β (potential)	Not defined for PCSK6. But basic cleavage site in the precursor for furin, commonly reported as R-H-R-R↓	Furin-mediated precursor processing is supported by biochemical and cellular experiments.	Furin	[3, 152] .
Propeptide of Nodal	RQRR↓-containing processing site.	Cell-based precursor-processing studies and embryonic developmental studies.	Furin and PCSK6	[32]
Propeptide of BMP	BMP4 has basic processing sites, including RSKR↓; processing may occur at more than one site.	Purified precursor/proprotein-convertase experiments, transfected-cell studies and embryonic models.	Furin, PCSK5, PCSK6 and PCSK7	[32, 153]
Propeptide of GDF15	RXXR↓-containing site in the propeptide, commonly reported as RRAR↓	Recombinant protein/peptide assays, transfected cells, cardiomyocytes and mouse models.	Furin, PCSK5 and PCSK6.	[33]
Propeptide of IGF-II	Not established.	Cell-based processing and skeletal-muscle differentiation experiments.	PCSK6	[40]
Propeptide of PDGF-A	Pro-PDGF-A is cleaved at the RRKR(86↓) site.	Cell based PDGF-A precursor processing experiments.	Furin, PCSK5 and PCSK6	[45]
Propeptide of PDGF-B	pro-PDGF-B at the RGRR(81↓) site	Cell based PDGF-A precursor processing experiments.	Furin, PCSK5, PCSK6 and PCSK7.	[46]
Propeptide of VEGF-C (potential)	Not established.	Biochemical and cellular studies of VEGF-C maturation	Furin, PCSK5 and PCSK7.	[49, 50]

Propeptide of IRB	Activation site around Arg735 in human IR-B; numbering depends on isoform and convention.	Furin-deficient cells, receptor maturation assays, convertase addition and inhibition.	Furin, PCSK5 and PCSK6	[41]
Propeptide of PAR-1	At the non-canonical site Arg46↓	Transfected cells, cleavage-site mutagenesis, receptor signaling assays.	Furin, PCSK5 and PCSK6.	[65]
PRR	RXXR↓	Recombinant peptide cleavage, mass spectrometry, prostate cancer cell knockdown and overexpression	PCSK6 and furin.	[80]
Propeptide of MMP2,	At RKPR↓ in the prodomain	Co-expression with convertases; site-directed mutagenesis; enzymatic assays; convertase inhibition.	Furin, PCSK5 and PCSK6.	[57, 154]
Propeptide of MMP9	Not established.	Pro-MMP9 and furin co-expression in HEK293E cells; site-directed mutation of the furin recognition motif; immunoblot detection of precursor processing.	Furin.	[155]
Propeptide of MMP14 (MT1-MMP)	At R108RKR111↓Y112	Convertase co-expression and cell-based processing assays; peptide-site cleavage assays; site accessibility analyses; studies of intracellular processing and cell-surface levels of MT1-MMP.	Furin, PCSK5 and PCSK7.	[156, 157]
Propeptide of MMP15 (MT2-MMP)	Not established.	Recombinant pro-MT2-MMP cleavage and N-terminal sequencing.	Furin.	[158]
Propeptide of MMP24 (MT5-MMP)	RRRNKR↓ in the N-terminal prodomain.	PCSK6 knockdown; co-expression and cleavage assays in cells; mutation or deletion of the recognition motif; in-vivo PCSK6 knockdown in an	Furin and PCSK6	[56]

		Alzheimer’s disease mouse model.		
Propeptide of ADAMTS-4	Arg212↓, within the RAKR sequence; additional processing sites have been reported.	Recombinant pro-ADAMTS-4, biochemical assays, human osteoarthritic cartilage and cytokine-stimulated cartilage.	Furin, PCSK5 and PCSK6.	[73, 74]
Propeptide of ADAMTS-5	Arg261↓, within an arginine-rich sequence.	Furin-deficient cells, convertase expression/inhibition, aggrecan degradation assays and cartilage studies.	Furin, PCSK6 and PCSK7.	[74, 159]
EL	Cleavage at RNKR330↓ releases inactive fragments and reduces EL lipase activity.	Co-expression with convertases; site-directed mutagenesis; N-terminal sequencing; convertase inhibitors; hepatocytes and mouse models.	Furin, PCSK5, PCSK6 and PCSK7.	[85, 87, 160]
LPL	After position 324, the second arginine in the RAKR sequence.	Cell-based expression and cleavage assays; co-expression with convertases; studies of ANGPTL3-mediated enhancement of LPL cleavage and loss of LPL activity.	Furin, PCSK5 and PCSK6.	[86, 87]
Propeptide of E-cadherin (potential)	Prodomain cleavage site; an exact PCSK6-specific bond is not established.	Cell-based processing assays and mouse embryonic development models.	Furin, PCSK6 and PCSK7.	[59]
Propeptide of Corin	Arg801↓	Purified PCSK6, HEK293 and HL-1 cells, R801A mutation, PCSK6 knockdown/overexpression and mouse models.	PCSK6.	[69, 70]

Abbreviations: BMP, mature bone morphogenetic protein; TGF-ß, transforming growth factor-ß; GDF15, growth differentiation factor 15; IGF-II, insulin-like growth factor-II; PDGF-A/B, pro-platelet-derived growth factor-A/B; VEGF-C, vascular endothelial growth factor-C; IRB, insulin receptor B; PAR-1, protease-activated receptor 1; PRR, prorenin receptor; MMP, matrix metalloproteinase; MT-MMP, membrane-type matrix metalloproteinase; ADAMTS-4/5, a disintegrin and metalloproteinase with thrombospondin

motif-4/5; EL, endothelial lipase; ANGPTL3, angiopoietin-like-3; LPL, lipoprotein lipase; E-cadherin, epithelial cadherin.
